# Lysis of Staphylococcal Cells by Modular Lysin Domains Linked via a Non-covalent Barnase-Barstar Interaction Bridge

**DOI:** 10.3389/fmicb.2019.00558

**Published:** 2019-03-22

**Authors:** Linnea C. Hjelm, Johan Nilvebrant, Per-Åke Nygren, Anders S. Nilsson, Johan Seijsing

**Affiliations:** ^1^Department of Protein Science, School of Engineering Sciences in Chemistry, Biotechnology and Health, KTH Royal Institute of Technology, Stockholm, Sweden; ^2^Department of Molecular Biosciences, The Wenner-Gren Institute, Stockholm University, Stockholm, Sweden

**Keywords:** endolysin, exolysin, barnase, barstar, fusion protein, non-covalent interaction, *Staphylococcus*, antibiotic alternative

## Abstract

Bacteriophage endolysins and bacterial exolysins are capable of enzymatic degradation of the cell wall peptidoglycan layer and thus show promise as a new class of antimicrobials. Both exolysins and endolysins often consist of different modules, which are responsible for enzymatic functions and cell wall binding, respectively. Individual modules from different endo- or exolysins with different binding and enzymatic activities, can via gene fusion technology be re-combined into novel variants for investigations of arrangements of potential clinical interest. The aim of this study was to investigate if separately produced cell wall binding and enzyme modules could be assembled into a functional lysin via a non-covalent affinity interaction bridge composed of the barnase ribonuclease from *Bacillus amyloliquefaciens* and its cognate inhibitor barstar, known to form a stable heterodimeric complex. In a proof-of-principle study, using surface plasmon resonance, flow cytometry and turbidity reduction assays, we show that separately produced modules of a lysin cysteine/histidine-dependent amidohydrolase/peptidase (CHAP) from *Staphylococcus aureus* bacteriophage K endolysin (LysK) fused to barnase and a cell wall binding Src homology 3 domain (SH3b) from the *S. simulans* exolysin lysostaphin fused to barstar can be non-covalently assembled into a functional lysin showing both cell wall binding and staphylolytic activity. We hypothesize that the described principle for assembly of functional lysins from separate modules through appended hetero-dimerization domains has a potential for investigations of also other combinations of enzymatically active and cell wall binding domains for desired applications.

## Introduction

In the exploration of antimicrobials to combat resistant bacterial infections, endolysins have gained attention as alternatives to conventional small molecular antibiotics (Schuch et al., 2014; Czaplewski et al., 2016; Jun et al., 2017; Totté et al., 2017). Endolysins are peptidoglycan hydrolases used by bacteriophages to release replicated progeny by degrading the cell wall of their bacterial host, resulting in cell lysis and death. Endolysins are functionally and structurally similar to both bacterial exolysins and autolysins. The exolysins are produced by certain bacteria with the function of causing lysis of alien bacterial species (Schindler and Schuhardt, 1964), and autolysins regulate cell wall metabolism and population size (Rogers et al., 1980). Development of resistance to endolysins is considered unlikely since these enzymes cleave evolutionary conserved and essential structures in the cell wall with high specificity for particular bacteria (Pastagia et al., 2011; Rodríguez-Rubio et al., 2013). At present, there are four endolysin-based products in ongoing clinical trials against staphylococcal infections (Totté et al., 2017), and one endolysin-based formulation has reached market approval as a medical device for topical application in treatment of less severe skin conditions such as rosacea, acne, and eczema (Herpers et al., 2014).

Endolysins need both to bind the surface of the target bacterium and to be able to cleave the peptidoglycan substrate. For endolysins targeting Gram-positive bacteria, these two functions are commonly mediated by separate domains referred to as cell wall binding domains (CBD) and enzymatically active domains (EAD). Moreover, individual endolysins may contain several EADs (Diaz et al., 1990). The modular properties of endolysins has inspired protein engineering efforts to find new combinations of EADs and CBDs with, from a pharmacological point of view, improved properties like altered strain specificity and/or higher catalytic efficacy (Croux et al., 1993; Schmelcher et al., 2011). Searches for effective new EAD-CBD combinations from collections of individual moieties may, however, involve construction of hundreds of gene fusions that need to be expressed individually before evaluation (Yang et al., 2015, 2017; Verbree et al., 2017). Thus, more effective means for linking sets of EADs and CBDs into various combinations for evaluation of their efficacies to lyse bacteria are called for.

**FIGURE 1:**
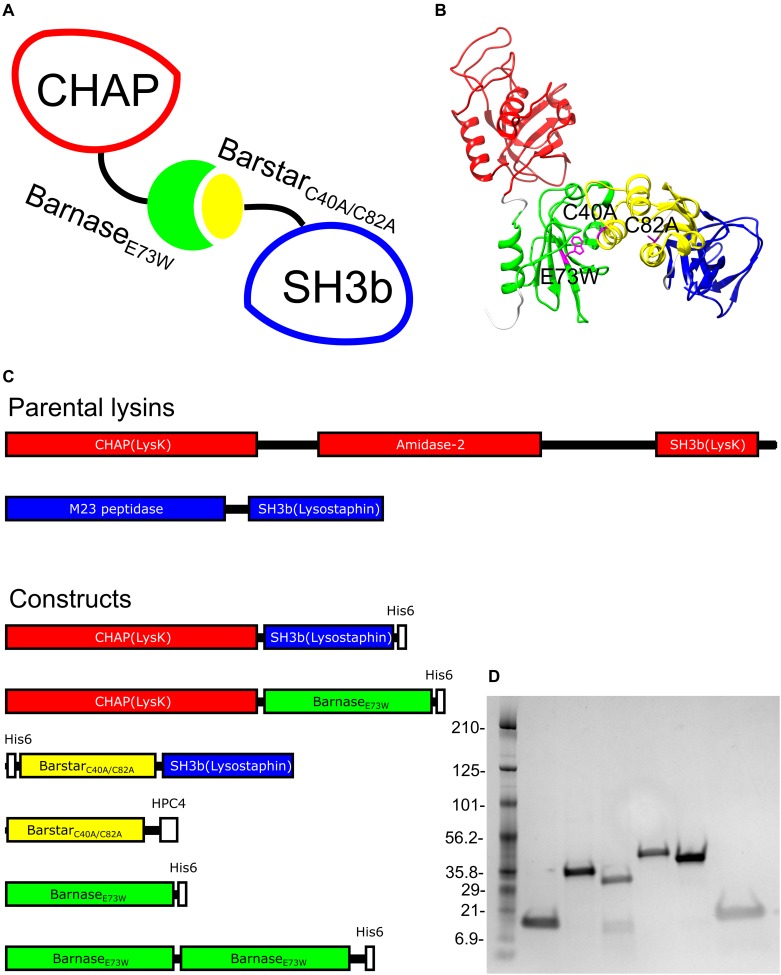
**(A)** Schematic sketch of protein complex, **(B)** structure prediction of complex made using I-TASSER (Zhang, 2008) with mutations highlighted in magenta. **(C)** Representations of parental lysins and protein constructs designed for this study. The parental endolysin LysK and the derived CHAPK domain is in red. The parental Lysostaphin and its derived SH3b domain are in blue. Barnase and barstar are in green and yellow, respectively. **(D)**. SDS–PAGE of protein constructs. From left to right: ladder, Barnase_E73W_, Barnase_E73W_-Barnase_E73W_, Barstar_C40A/C82A_-SH3b, CHAP-Barnase_E73W_, CHAP-SH3b, and Barstar_C40A/C82A_.

Barnase is a small *Bacillus amyloliquefaciens* ribonuclease of 110 amino acids showing a high affinity to its own inhibitor, the 89 amino acid barstar (Hartley, 1993; Schreiber and Fersht, 1993). The strong interaction between barnase and barstar has previously been exploited to achieve both stoichiometrically and structurally ordered assemblies of designed multivalent antibody-fragment protein complexes (Deyev et al., 2003). In the present study, we have investigated the possibility to utilize the barnase-barstar system in a modular approach for non-covalent, yet robust, linking of EADs and CBDs into functional cell wall binding and enzymatically active lysins (Figure 1A,B). We report the results from endolysin assembly of the EAD cysteine/histidine-dependent amidohydrolase/peptidase (CHAP) from *Staphylococcus aureus* bacteriophage K endolysin (LysK) with the CBD Src homology 3 domain (SH3b) from the *S. simulans* exolysin lysostaphin.

## Results and Discussion

### Design, Production and Biochemical Characterization of CHAP-Barnase_E73W_ and Barstar_C40A/C82A_-SH3b Fusion Proteins

Due to the RNAse activity of wild type barnase, which has been described to be toxic to bacterial cells used for production, a previously described enzymatically inactive variant (E73W) of barnase was used as fusion partner for the construction of a CHAP-Barnase_E73W_ fusion protein (Hartley, 1993; Ramachandran and Udgaonkar, 1996; Figure 1A–C). For construction of the cognate Barstar-SH3b fusion protein, a double cysteine mutant (C40A/C82A) of barstar was used to avoid potential problems with artefactual disulfide bridges between protein constructs, potentially interfering with the interpretation of the results (Hartley, 1988; Schreiber et al., 1997; Khait and Schreiber, 2012). Constructs for prokaryotic expression of the CHAP-Barnase_E73W_ and Barstar_C40A/C82A_-SH3b lysin modules were assembled (Figure 1C). For use as controls, expression constructs for the single domains of Barstar_C40A/C82A_ and Barnase_E73W_, a Barnase_E73W_-Barnase_E73W_ dimer to be used as a non-sterically hindered ligand in surface plasmon resonance measurements and a CHAP-SH3b direct fusion protein were also assembled.

In initial constructs, a GGGS linker was used to connect the domains in the different fusion proteins. However, after expression of protein constructs containing CHAP it was realized that this endopeptidase, known to cleave between the pentaglycine and the D-alanine of the staphylococcal peptidoglycan (Becker et al., 2009), was also able to degrade the GGGS linker (data not shown). Thus, the linker in fusion constructs containing CHAP was changed into GSSG, which resulted in proteolytically stable constructs.

All proteins were produced as His_6_-tagged constructs intracellularly in *Escherichia coli* and purified from the soluble cytoplasmic fraction using immobilized metal ion affinity chromatography or, in the case of the single domain Barstar_C40A/C82A_ construct which was produced in fusion with a short HPC4 peptide (Rezaie et al., 1992), using an anti-protein C immunoaffinity column (Figure 1C). The purified proteins were analyzed by SDS–PAGE (Figure 1D), circular dichroism (Table 1) and mass spectrometry (Table 1).

**Table 1:** Melting temperatures, theoretical and experimental molecular weights of constructs.

Construct name	Theoretical molecular weight (Da)	Experimental molecular weight (Da)	Melting temperature (°C)
CHAP-SH3b	29 185	29 184^#^	41.0
CHAP-Barnase_E73W_	32 133	32 132^#^	42.5
Barstar_C40A/C82A_-SH3b	20 933	21 292^†^	41.0
Barstar_C40A/C82A_	12 597	12 609^†^	N/A
Barnase_E73W_	13 407	13 406^#^	N/A
Barnase_E73W_-Barnase_E73W_	26 969	26 968^#^	N/A


*The experimental molecular weights have been determined with MALDI-TOF(^†^) or LCMS(^#^). The melting temperature of samples marked N/A were not analyzed.*

### CHAP-Barnase_E73W_ Binds Barstar_C40A/C82A_-SH3b

**FIGURE 2:**
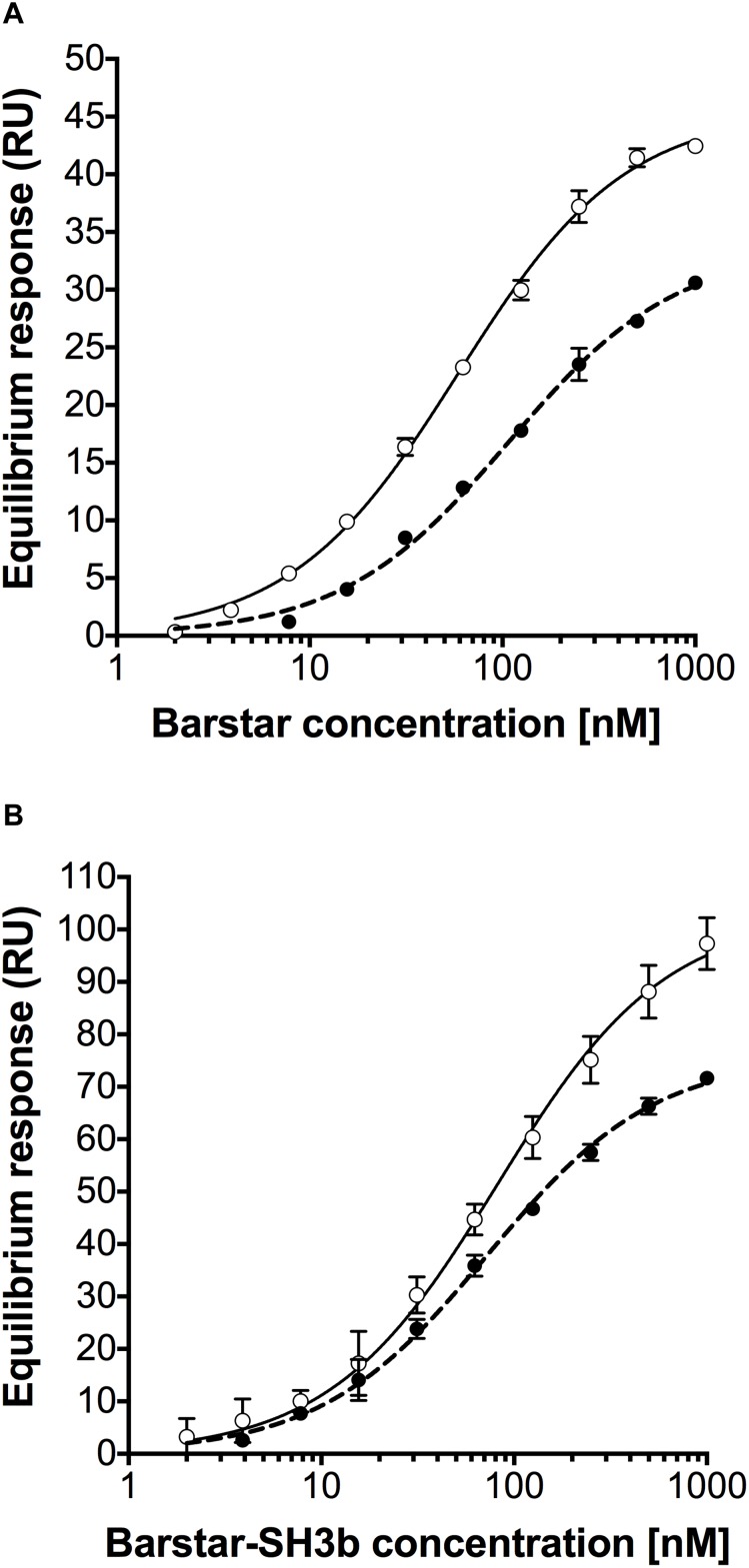
Equilibrium response curves from injection of **(A)** Barstar_C40A/C82A_ over Barnase_E73W_-Barnase_E73W_ surface (open circles), Barstar_C40A/C82A_ over CHAP-Barnase_E73W_ surface (black circles), **(B)** Barstar_C40A/C82A_-SH3b over Barnase_E73W_-Barnase_E73W_ surface (open circles), and Barstar_C40A/C82A_-SH3b over CHAP-Barnase_E73W_ surface (black circles). Error bars represent the standard error of the mean from two replicates.

The binding affinity between the barnase_E73W_ and the barstar_C40A/C82A_ domains of the different constructs was investigated using surface plasmon resonance (SPR) technology. Here, Barnase_E73W_-Barnase_E73W_ or CHAP-Barnase_E73W_ proteins were immobilized on the sensor chip followed by injection of Barstar_C40A/C82A_ or Barstar_C40A/C82A_-SH3b proteins at different concentrations. The equilibrium dissociation constants (K_D_) of the interactions were determined from observed equilibrium responses (Figure 2 and Table 2). The affinity between Barstar_C40A/C82A_ and Barnase_E73W_-Barnase_E73W_ (Figure 2A) was determined to 59 nM. The Barstar_C40A/C82A_-SH3b fusion protein (Figure 2B) displayed a similar affinity (82 nM) as Barstar_C40A/C82A_ suggesting that the interaction was not influenced by fusion to the SH3b moiety. Injections of Barstar_C40A/C82A_ (Figure 2A) or the Barstar_C40A/C82A_-SH3b fusion protein (Figure 2B) over immobilized CHAP-Barnase_E73W_ showed that these interactions were of similar strength (108 and 73 nM, respectively). Injection of the negative controls bovine serum albumin (BSA), human serum albumin (HSA) and the monoclonal IgG antibody trastuzumab gave no responses (data not shown). Taken together, these results indicate that fusion of the CHAP or SH3b domains did not have a significant influence on the interaction strength between the barstar_C40A/C82A_ and barnase_E73W_ domains. Neither did the fusions cause any detectable unspecific binding response.

**Table 2:** Affinities from surface plasmon resonance analyses (mean from two replicate series of measurements and standard deviation).

Analyte	Ligand	K_D_ (nM)
Barstar_C40A/C82A_-SH3b	Barnase_E73W_-Barnase_E73W_	82 ± 7
Barstar_C40A/C82A_-SH3b	CHAP-Barnase_E73W_	73 ± 4
Barstar_C40A/C82A_	Barnase_E73W_-Barnase_E73W_	59 ± 2
Barstar_C40A/C82A_	CHAP-Barnase_E73W_	108 ± 10


### Barstar_C40A/C82A_-SH3b Binds *Staphylococcus carnosus*

**FIGURE 3:**
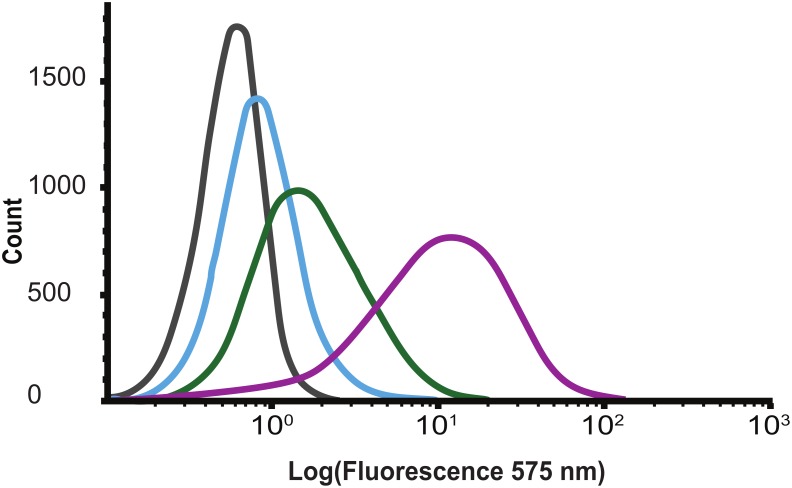
Flow cytometry histograms showing concentration dependent (gray 0, blue 110, green 330, and purple 1000 nM) binding of biotinylated Barstar_C40A/C82A_-SH3b to *Staphylococcus carnosus*.

Flow cytometry was used to investigate the cell wall binding properties of the Barstar_C40A/C82A_-SH3b fusion protein module. Here, different concentrations of biotinylated Barstar_C40A/C82A_-SH3b protein were incubated with Gram-positive *S. carnosus*. After washing, any cell-bound protein construct was detected using a streptavidin-phycoerythrin conjugate. The results showed that the Barstar_C40A/C82A_-SH3b fusion protein bound to *S. carnosus* in a concentration-dependent manner (Figure 3). This confirms that the recombinantly produced SH3b domain is able to bind to the Staphylococcus peptidoglycan layer (Gründling et al., 2006) and retains its binding after fusion to the barstar_C40A/C82A_ moiety. In control experiments, *E. coli* cells were used and yielded no signal shift (Supplementary Figure S1) indicating no unspecific binding to outer membrane components of the surface of Gram-negative *E. coli* cells.

### The Assembled CHAP-Barnase_E73W_/Barstar_C40A/C82A_-SH3b Lysin Shows Staphylolytic Activity

**FIGURE 4:**
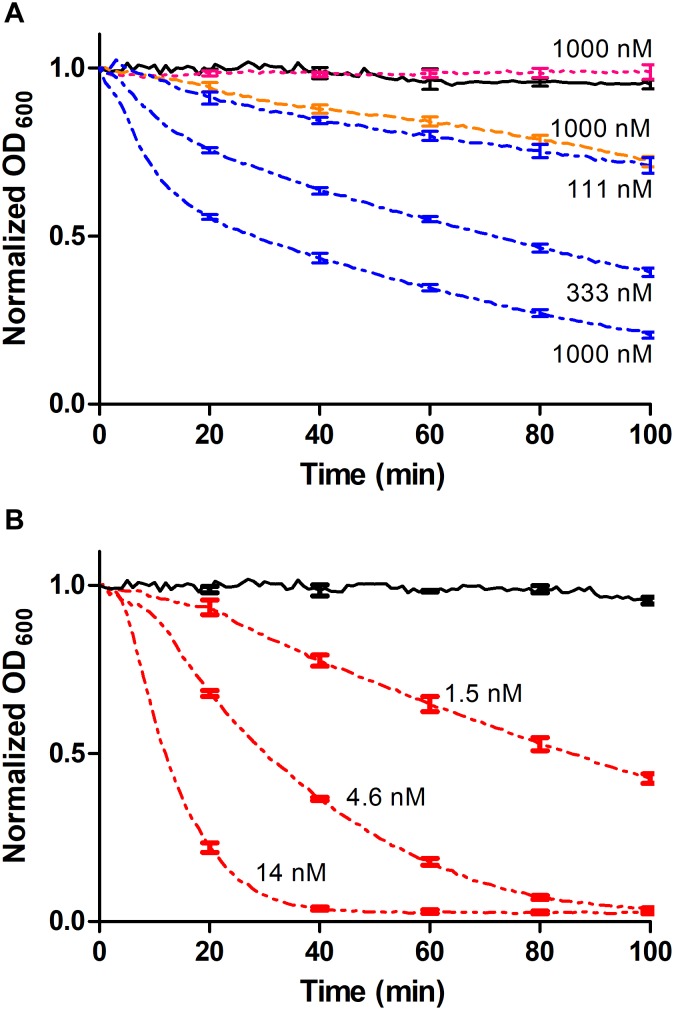
Turbidity reduction assays (TRA). Data points were collected every minute and error bars representing standard deviation is shown each 20 min for intelligibility. **(A)** The CHAP-BarnaseE73W/BarstarC40A/C82A-SH3b complex was used at concentrations of 1000, 333, and 111 nM, respectively (blue dashed/dotted line). The non-complexed controls Barstar_C40A/C82A_-SH3b (cyan dotted line) and CHAP-Barnase_E73W_ (orange dashed line) were used at a concentration of 1 μM and PBS (black continuous line) was used as negative control. **(B)** The CHAP-SH3b gene fusion had a concentration of 14, 4.6, and 1.5 nM, respectively (red dashed/dotted/dotted line) and PBS (black continuous line) was used as negative control.

To investigate if a complex between the CHAP-Barnase_E73W_ and Barstar_C40A/C82A_-SH3b modules could result in a functional lysin, a turbidity reduction assay (TRA) involving *S. carnosus* cells was performed in which the individual Barstar_C40A/C82A_-SH3b and CHAP-Barnase_E73W_ modules were used as controls. As expected, no reduction of the turbidity was observed when cells were incubated with the Barstar_C40A/C82A_-SH3b module alone whereas a low reduction of the turbidity was observed for the CHAP-Barnase_E73W_ construct (Figure 4A and Table 3). This is in agreement with previously reported lytic effects of CHAP from LysK also in the absence of a CBD (Horgan et al., 2009). However, when cells were incubated with the heterodimerized CHAP-Barnase_E73W_/Barstar_C40A/C82A_-SH3b complex, a significantly enhanced and concentration-dependent reduction in turbidity was observed (Figure 4A and Table 3). For comparison in the experiment, the CHAP-SH3b gene fusion reference construct was included. As could be expected, the cell lysis capacity observed for this covalently linked construct was considerably higher. Nevertheless, the fact that a cell lysis effect was seen for the non-covalently linked CHAP-Barnase_E73W_/Barstar_C40A/C82A_-SH3b complex holds promise that the barnase/barstar-based system for modular lysin assembly has a potential to be used for qualitative assessment of the compatibility also of other individual EADs and CBDs. Once compatible combinations are identified, direct gene fusion between these could provide leads for further studies, including pre-clinical and clinical tests.

**Table 3:** Specific activities of individual protein constructs and non-covalent protein complexes (mean and standard deviations from triplicate measurements).

Construct/Complex	Specific activity [ΔOD_600_/(min^∗^μM)]
CHAP-SH3b	3.6 ± 0.19
Barstar_C40A/C82A_-SH3b	ND
CHAP-Barnase_E73W_/Barstar_C40A/C82A_-SH3b complex	0.017 ± 0.00059
CHAP-Barnase_E73W_	0.00081 ± 0.00010


The described modular assay concept would potentially benefit from investigating different linker types and lengths between included moieties and a stronger affinity between the appended dimerization domains to increase the fraction of modules being in complex to each other at a given concentration. For instance, in the TRA data presented in Figure 4A, the concentrations of the individual modules used were in the range 111 nM to 1000 nM, corresponding to approximately 1.5–14 times the equilibrium dissociation constant. In this concentration range only 45–76% of the individual modules could under ideal conditions be expected to be present in heterodimeric complexes, limiting the assay sensitivity. By mutating barnase_E73W_ and/or barstar_C40A/C82A_ back to wild type, a significantly higher affinity between the modules could be expected, although this would be associated with difficulties during recombinant production due to toxicity from the barnase activity (Hartley, 1989). However, it has been shown to be possible to produce also the wild type barnase in *E. coli*, if co-expressed with the barstar inhibitor and having it removed by denaturation *in situ* during IMAC purification (Deyev et al., 2003). Alternatively, other cognate fusion partners as leucine zippers (Kostelny et al., 1992), anti-idiotypic affibody molecules (Eklund et al., 2002; Lindborg et al., 2013), or different fusion partner pairs capable of forming a covalent bond between themselves after binding, including intein moieties (Shah and Muir, 2014) or the SpyCatcher/SpyTag domains (Zakeri et al., 2012) could be used. Enzymatic coupling of separately produced modules using the transpeptidase sortase may also be considered (Mao et al., 2004).

Alternative means to screen for novel chimeric EAD-CBD combinations with desired lytic activities have been described, based on combinatorial assembly of lysin modules at the genetic level, rather than on the protein level as in the present study. Here, host cells expressing the different combinations are lysed enzymatically or chemically to release the lysin for assessment of the activity toward the investigated target bacterial cells, after which hits are identified via DNA sequencing (Yang et al., 2015, 2017; Verbree et al., 2017). Compared to the addition of controlled amounts of pre-purified lysins to target cells, these approaches may experience biases from variations in expression levels between different chimeras and a more limited control of the exact assay conditions. Nevertheless, such approaches are very interesting and add to the available means for investigating novel EAD-CBD combinations.

In conclusion, the described experiments show that CHAP and SH3b modules can be physically linked through the non-covalent barnase-barstar interaction and that formed complexes retain both the cell wall binding and the staphylolytic activity of the separate modules. Although only showed for one specific case, the results demonstrate for the first time the interesting principle that a functional lysin can be assembled from separately produced and affinity domain-tagged cell wall binding and catalytic modules, respectively. This holds promise for investigations of other EAD and CBD lysin modules using similar principles.

## Materials and Methods

### Chemicals and Reagents

All chemicals and reagents were bought from Sigma-Aldrich if not otherwise stated.

### Cloning and Mutagenesis

DNA constructs were synthesized and cloned into pET-24a(+) or pET-26b(+) vectors (Merck, Darmstadt, Germany) by BioCat (Heidelberg, Germany) or BioBasic (Markham, ON, Canada). Site directed mutagenesis was performed by Biozilla (Sacramento, CA, United States).

### Structure Prediction of Fusion Proteins

Amino acid sequences of the two fusion proteins CHAP-Barnase_E73W_ and Barstar_C40A/C82A_-SH3b were uploaded and run with standard settings in the protein structure predictor I-TASSER (Zhang, 2008). Returned predicted structures were aligned to the barnase/barstar complex (2ZA4) using the PyMOL Molecular Graphics System (Version 1.3, Schrödinger, LLC) and secondary structure elements were corrected to what has previously been seen in the barnase/barstar complex, LysK, and Lysostaphin (2ZA4, 4CSH, 5LEO) using UCSF Chimera 1.11.2 (Pettersen et al., 2004; Urakubo et al., 2008; Sanz-Gaitero et al., 2014).

### Recombinant Expression and Purification

Plasmids were transformed into BL21 (DE3) competent *E. coli* cells (Merck, Darmstadt, Germany), and grown to an OD_600_ of 0.6 after which the culture was cooled on ice and induced with 0.5 mM IPTG (ThermoFisher Scientific, Waltham, MA, United States). Protein expression was performed overnight at 20°C and 200 rpm.

Protein expressing bacteria were pelleted using centrifugation, resuspended in lysis buffer [50 mM NaH_2_PO_4_, 300 mM NaCl, 10 mM imidazole, 30% glycerol, pH 8.0], homogenized using an EmulsiFlex-C3 (Avestin, Mannheim, Germany) and sonicated by a Vibra-Cell VCX 130 sonicator (Sonics, CT, United States). Cell debris was removed by centrifugation and lysates loaded on His GraviTrap columns (GE Healthcare, Uppsala, Sweden). Columns were washed with lysis buffer and protein eluted with elution buffer [50 mM NaH_2_PO_4_, 300 mM NaCl, 250 mM imidazole, 30% glycerol, pH 8.0]. The monomeric construct Barstar_C40A/C82A_ with a HPC4-tag was purified on Anti-Protein C Affinity Matrix (Roche, Penzberg, Upper Bavaria, Germany) according to the manufacturer’s instructions. Size and purity of the produced protein constructs was verified using Mini-Protean TGX Gels (Bio-Rad, Hercules, CA, United States) and SCIEX 4200 MALDI-TOF (SCIEX, Framingham, MA, United States) or LCMS mass spectrometry (UltiMate 3000, ThermoFisher Scientific, Waltham, MA, United States; Impact II, Bruker, Billerica, MA, United States) according to manufacturer’s instructions.

### Circular Dichroism Spectroscopy

CHAP-SH3b, CHAP-Barnase_E73W_ and Barstar_C40A/C82A_-SH3b were buffer exchanged to PBS and diluted to a final concentration of 0.3–0.5 mg/ml for analysis by circular dichroism. Using the Chirascan system (Applied Photophysics, Surrey, United Kingdom) with a 1 mm High precision cell (110-1P-40 cuvettes, Hellma Analytics, Germany). Ten wavelength scans were recorded between 190 and 280 nm at 20°C. All analyzed constructs gave signal at 210 nm and this wavelength was used to determine the melting point using a temperature gradient of 1°C/min.

### Surface Plasmon Resonance

Proteins were buffer exchanged to PBST [150 mM NaCl, 8 mM Na_2_HPO_4_, 2 mM NaH_2_PO_4_, 0.005% Tween 20, pH 7.4] on PD10 columns (GE Healthcare, Uppsala, Sweden) and filtered prior to SPR analysis using a Biacore T200 system (GE Healthcare, Uppsala, Sweden) at 25°C. Concentrations of the constructs were determined by absorbance measurements.

Approximately 700 RU of Barnase_E73W_-Barnase_E73W_, and CHAP-Barnase_E73W_ dissolved in 10 mM NaOAc pH 4.5 were immobilized by amine coupling in individual flow cells on a CM5 Series S sensor chip (GE Healthcare, Uppsala, Sweden). Barstar_C40A/C82A_ and Barstar_C40A/C82A_-SH3b were injected in duplicates using two-fold dilution series, spanning between 2 and 1000 nM at 30 μl/min with an association time of 500 and 1000 s dissociation time. Surfaces were regenerated between samples with two short (30 s) pulses of 10 mM HCl. Three control samples were injected at 1000 nM; HSA (Sigma-Aldrich, St. Louis, MO, United States), BSA (New England Biolabs, Ipswich, MA, United States) and the monoclonal antibody trastuzumab (Apoteket AB, Stockholm, Sweden).

Sensorgrams were double referenced toward the blank surface and a buffer injection. Report points were collected at the end of each injection for affinity calculations. In contrast to low analyte concentrations, responses at high concentrations did not level off at an equilibrium level in the end of the injections. This effect, probably caused by unspecific binding, gave a concentration-dependent linear contribution to the response curves, which was calculated and subtracted using GraphPad Prism (Version 5, San Diego, CA, United States). Equilibrium dissociation constants (K_D_) were calculated from plots of log(c) versus adjusted response signals.

### Flow Cytometry

The specific binding of biotinylated Barstar_C40A/C82A_-SH3b to the cell wall of *S. carnosus* TM300 was evaluated by using a Gallios flow cytometer (Beckman Coulter, Brea, CA, United States). Barstar_C40A/C82A_-SH3b was labeled with EZ-Link NHS-LC-LC-Biotin (ThermoFisher Scientific, Waltham, MA, United States) according to manufacturer’s instructions. Successful biotinylation was verified by capture on streptavidin-coated magnetic beads (Dynabeads M280, ThermoFisher Scientific, Waltham, MA, United States) followed by analysis by SDS–PAGE (Bio-Rad Laboratories, Hercules, CA, United States) as well as on an SCIEX 4200 MALDI-TOF Mass Spectrometry system (SCIEX, Framingham, MA, United States) (data not shown). Bacteria for analysis were grown in Terrific Soy Broth supplemented with yeast extract (TSB+Y) overnight at 37°C, 150 rpm. The OD_600_ of the cultures was used to normalize the bacterial amount between experiments. Cells were pre-washed twice in PBS supplemented with 0.1 % (w/v) Pluronic F 108 NF Prill Poloxamer (PBS-P) before incubating with the biotinylated Barstar_C40A/C82A_-SH3b at the final concentration of 110, 330, or 1000 nM. Following the 45 min long incubation at room temperature, samples were washed twice and resuspended in Streptavidin R-Phycoerythrin conjugate solution (SA-PE; 0.5 μg/ml, #S866, ThermoFisher Scientific; Waltham, MA, United States) and continued to be incubated for 20 min in the dark and on ice. Cells were washed once and resuspended in cold PBS-P prior to flow-cytometric analysis. Fluorescent signal from the R-phycoerythrin was recorded for 200,000 cells per sample at 575 nm. The experiment was performed in duplicate and the data were analyzed using Kaluza (Version 2.1, Beckman Coulter, Brea, CA, United States). Controls included *S. carnosus* cells only, *S. carnosus* cells incubated with; biotinylated Barstar_C40A/C82A_-SH3b, secondary reagent (SA-PE), or Barstar_C40A/C82A_ and secondary reagent (SA-PE). As well as a negative control composed of *E. coli* (BL21) cells and *E. coli* (BL21) cells stained with 1000 nM Barstar_C40A/C82A_-SH3b and secondary reagent (SA-PE).

### Turbidity Reduction Assay

*Staphylococcus carnosus* TM300 substrate cells were grown to an OD_600_ of 0.4 as previously described by Schmelcher et al. (2012). The cells were pelleted, washed twice in cold buffer [10 mM Na_2_HPO_4_, 150 mM NaCl, 25% glycerol, pH 7.5], resuspended and stored at -80°C.

Triplicates of substrate cells and lysin modules were prepared in PBS in a 96-well plate to a total volume of 200 μl. Final OD_600_ was equal to 1 for the substrate cells and concentrations of the lysin modules ranged from 0.15 to 1000 nM in three-fold dilution steps. PBS was used as negative control. Reduction in OD_600_ was monitored over time at room temperature using a POLARstar Omega plate reader (BMG Labtech, Cary, NC, United States) and used to calculate the enzymatic activity.

The specific enzymatic activities of the individual constructs and the non-covalent complexes were calculated. First, the steepest slope [ΔOD_600_/min] of the lysis curves was identified in sigmoid [A], exponential [B] or linear [C] functions to best fit the lysis data. The slope of the PBS controls was subtracted. The resulting values were plotted against the respective construct concentration and function [D] was fitted to the emerging curve. The specific enzymatic activity, namely the slope [ΔOD_600_/(min^∗^μM)] of the curve in origo, was calculated as [k ^∗^a] of function [D].

$$[A]f=a+\frac{b-a}{1+e^{-c*\left(time-d\right)}}$$

$$[B]f=a+b*e^{-c*time}$$

[C]f =a*time+b

$$[D]f=a*\left(1-e^{-k*x}\right)$$

## Data Availability

All datasets generated for this study are included in the manuscript and/or the Supplementary Files.

## Author Contributions

LH, JN, P-ÅN, and JS conceived and designed the experiments. LH and JS performed the experiments. LH, JN, P-ÅN, AN, and JS analyzed the data. JN contributed to reagents, materials, and analysis tools. LH, JN, P-ÅN, AN, and JS wrote the manuscript. AN and JS provided the funding.

## Conflict of Interest Statement

The authors declare that the research was conducted in the absence of any commercial or financial relationships that could be construed as a potential conflict of interest.
