# Supplementary material for: Lysis of Staphylococcal Cells by Modular Lysin Domains Linked via a Non-covalent Barnase-Barstar Interaction Bridge
Source: Front Microbiol. 2019 Mar 22;10:558. doi: 10.3389/fmicb.2019.00558 (PMC6439198; doi:10.3389/fmicb.2019.00558)
Supplement: Supplementary file 1 [file Data_Sheet_1.docx]

Supplementary Material


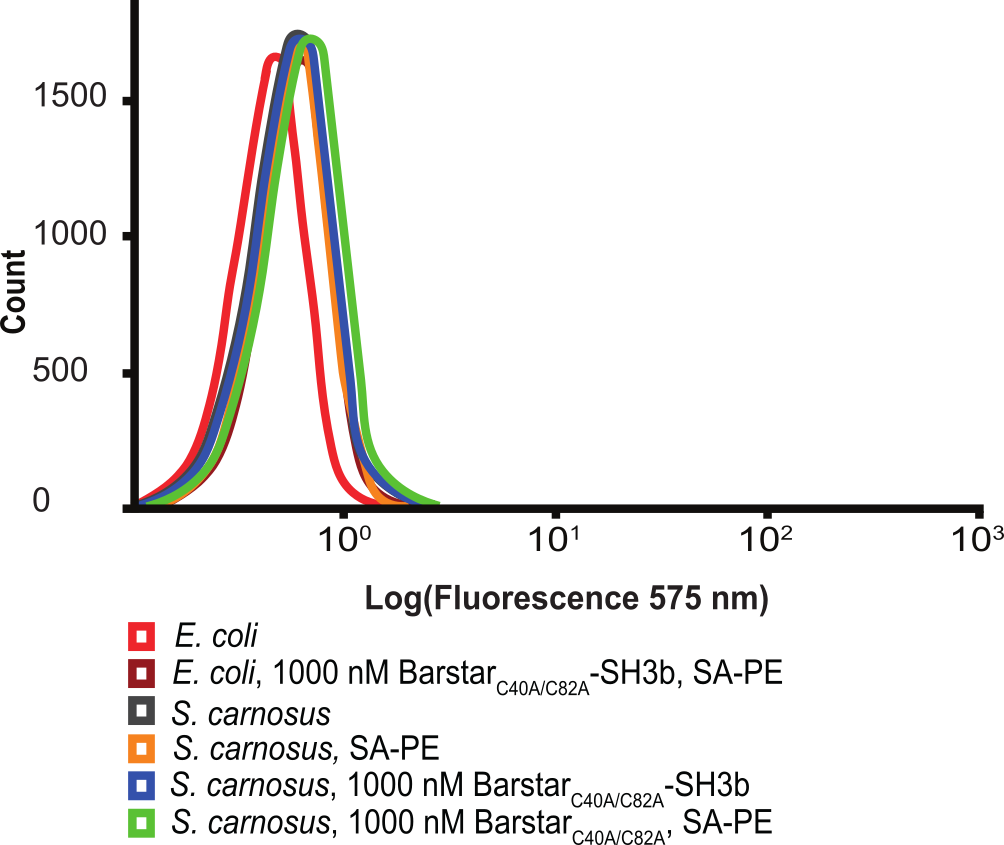


**Figure S1 |** Flow cytometry histograms of controls for the bacteria *S. carnosus* and *E. coli*.
